# Presenting Eco-Anatomical Data for *Saponaria jagelii*, a Species on the Edge of the Blade

**DOI:** 10.3390/life14030398

**Published:** 2024-03-17

**Authors:** Aikaterina L. Stefi, Konstantina Mitsigiorgi, Nikolaos S. Christodoulakis

**Affiliations:** Section of Botany, Department of Biology, Faculty of Sciences, National and Kapodistrian University of Athens, 15701 Athens, Greece; mitsig@biol.uoa.gr (K.M.); nchristo@biol.uoa.gr (N.S.C.)

**Keywords:** endemic plant, critically endangered plant, plant anatomy, seeds, *Saponaria jagelii*

## Abstract

The seeds, roots, leaves, flowers and fruits of the critically endangered (CR) species *Saponaria jagelii* Phitos & Greuter (Caryophyllaceae) were studied. The morphology of the seeds was investigated with scanning electron microscopy. The seeds were imbibed, germinated and developed into young plants. These plants, along with strictly selected wild-growing plants, were used for optical microscopic observations. The leaves and flowers were observed with scanning electron microscopy as well. At least two types of active glandular trichomes were detected on both the leaves and the calyxes of the flowers. The structures of the primary and secondary roots were also investigated. The roots turned into secondary structures very quickly and very close to the root tip. Light microscopy and histochemical reagents were employed to detect secondary metabolites of interest in the leaves. All the metabolites detected were already reported to be synthesized in stressed plants. Distribution data are presented. Conservation actions based on the habitat morphology and the human activities within it, such as the limitation of beach access during the seed-dispersing period and the prohibition of vehicle usage, are recommended in order to protect this tolerant yet severely stressed plant species.

## 1. Introduction

*Saponaria jagelii* Phitos & Greuter (Caryophyllaceae) is a small, annual, narrow endemic species of Greece, with an erect, robust 3–10 cm long branching stem, thriving only on two scattered, very restricted localities in the Western part of the small island of Elafonisos (25 km^2^) [1]. This island is located 600 m away from the southern coast of the Peloponnese. It has been reported, though without confirmation, that the species also exists on the Malea peninsula (South Peloponnese). Recently, the species has also been reported to exist on the sand dunes located on the south-east coast of the island of Limnos (North Aegean Sea), but no detailed data were published [2].

The name of the genus *Saponaria* derives from the Latin word *sapo*-*saponis*, referring to soap, indicating the use of some species of the genus in soap making. According to the Flora of Greece, only eight species of the genus are found in Greece. Two of them are Greek endemic: *Saponaria jagelii* and *Saponaria aenesia*. The latter is endemic to the island of Kefallinia, on Mount Ainos. The other endemic species, *Saponaria jagelii*, was named after A. Jagel, the student who first collected this species [1].

**Figure 1:**
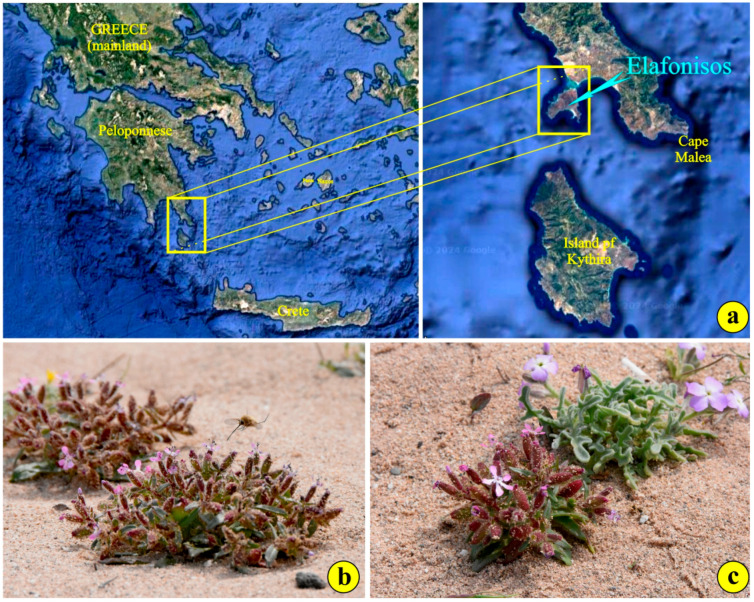
(**a**) The broad distribution area of *Saponaria jagelii* is the island of Elafonisos (©Google Maps, Imagery©2024 Airbus, CNES); (**b**) an individual is visited by the specific pollinator *Bombylius* sp.; (**c**) *Saponaria jagelii* and *Matthiola tricuspidata* are common neighbors.

The plants grow exclusively in EU priority habitat 2120—along the shoreline with *Ammophila arenaria* (white dunes)—in the NATURA2000 site GR2540002 “Periochi Neapolis kai Nisos Elafonisos” (Figure 1a). Their stems are reddish and covered with scattered glandular trichomes in the upper part [1]. The lower part is rather smooth. The leaves are fleshy and reddish-green, 1–4.5 cm long and lanceolate (Figure 1b). The leaf margins occasionally display delicate trichomes (Figure 1b). The upper leaf surface and leaf stalks present thick pubescence as well. The calyx is cylindrical and reddish, bears short teeth and is covered with glandular trichomes (Figure 1b,c) [1]. The petals are purple and tapered toward the base (Figure 1c) [1]. The flowering period extends from the end of March until early May, while the fruiting season extends from early May until early June. Dispersal occurs shortly after when the fruit, a nearly cylindrical capsule, opens and releases the seeds.

The species seems to be a part of the typical plant community growing in disturbed areas along sand dunes [1,3,4,5]. The most frequent “neighbors” of *S. jagelii* are *Euphorbia paralias* L., *Ammophila arenaria*, *Pancratium maritimum* (with greater spatial spread than a year ago), *Matthiola tricuspidata* (Figure 1c), *Medicago marina* L., *Silene sedoides* Poir., *Centaurea raphanina* subsp. *mixta* and *Anagallis arvensis*.

The species has been categorized as CR (critically endangered) according to IUCN Red List Criteria B1ab (i, ii, iii, v) + B2ab (i, ii, iii, v) [4] because it is known to occur only at two sites covering a very small area on two restricted sandy seashores. Furthermore, the quality of the habitat and the number of individuals is expected to decline.

Tourism is rapidly developing on the island, and several human activities on the beaches, such as the transit of motor vehicles and trampling by visitors, represent major threats, especially during the flowering period (threat 1.3: Tourism and recreation areas; threat 6.1: Recreational activities) [6]. Finally, the introduction of alien, invasive species (*Aptenia cordifolia* and *Carpobrotus edulis*) from neighboring private property also imposes extra pressure on the species for their survival.

Moreover, to the best of our knowledge, no single piece of information is available for the anatomical features of the leaves, stems, roots, flowers and seeds or for the germination ability of this critically endangered plant. It seems that determining the optimal conditions for seed germination will facilitate the *ex-situ* conservation of this species.

Concerning all the above, we launched this investigation intending to establish a detailed description of some structural and a few ecophysiological features to facilitate the conservation and *ex-situ* culture of a plant that, regrettably, has poor survival prospects in the second half of the twenty-first century [6].

## 2. Materials and Methods

Visits to the site

We approached the two restricted localities on the small island of Elafonisos in two consecutive years:

1st year

Visit: 25 May 2022, at the end of flowering season. Sampling: seeds were collected.

2nd year

Visit: 1–3 April 2023, mid-flowering season. Sampling: capsules and seeds were collected.

### 2.1. Plant Material

#### 2.1.1. Seed Germination

Intact capsules were collected *in-situ* during our visits to the habitat. They were placed in specific incubators (Heraeus B5050, West Midlands, UK) for desiccation at a constant temperature (17 °C). The humidity was gradually reduced. After three months, remnants of the pericarp were removed so that the seeds were completely clean, and they were transferred back to the incubator for 1 month. Seed germination took place in a P-Selecta incubator (Model No. 2000238, Barcelona, Spain) ventilated through a HAILEA ACO-9160 (China) at an output of 4 L/min. Then, they were transferred to culture chambers (elvem—model BOD100, GR) under controlled conditions (temperature/light), and their germination ability was tested at various temperatures (5, 10, 12.5, 15 and 20 °C). These seedlings, along with two individuals transferred from the habitat (see below), were fixed for microscopic observations. In addition, unsplit capsules were ruptured in the lab in order to count the number of seeds per capsule.

#### 2.1.2. Microscopy

During our first visit to the site (2–4 April 2022), we detached only two plants for further treatment. Small parts from the middle of the upper leaves, the roots close to the root tip and the stems were removed at random. Whole mounts of flowers and seeds were also obtained. The tissues were fixed in phosphate-buffered 3% glutaraldehyde (Merck KGaA, Darmstadt, Germany—pH 6.8) at 0 °C for 2 h and post-fixed in phosphate-buffered 1% osmium tetroxide (Merck KGaA, Darmstadt, Germany). They were dehydrated in graded ethanol series. The properly prepared pieces underwent one of the following:

(a) The pieces were transferred to 100% acetone, critical-point-dried (Autosamdri^®^-815, Tousimis, Rockville, MD, USA), double coated with gold and platinum and viewed with a JEOL JSM-6360 high-vacuum scanning electron microscope (Tokyo, Japan). All electron micrographs were taken with the instrument’s built-in camera (accelerating voltage 20 kV; spot size 50).

(b) The tissue, dehydrated in absolute ethanol, was transferred to propylene oxide and imbued in gradually increasing concentrations of Durcupan ACM (Fluka, Steinheim, Switzerland) (four-component epoxy resin). Finally, the tissue was left in pure Durcupan to polymerize at 70 °C for 36 h. Semithin sections obtained with glass knives from an LKB Ultrotome III (Sweden) were placed on glass slides and stained with 0.5 toluidine blue O (in 1% borax solution) as a general stain for light microscopic observations [7]. Sections of fresh or epoxy-embedded material were viewed with an OLYMPUS CX-41 light microscope (Japan). The original light micrographs were recorded digitally using a Nikon D5600 camera at 24.2 megapixels. The literature on double fixation was cited in detail by Christodoulakis et al. [8] and Christodoulakis et al. [9]. All micrographs of the leaves and flowers originate from tissues of the detached wild-growing plants. The root micrographs originate from the grown seedlings.

#### 2.1.3. Histochemistry

A histochemical investigation was executed using sections of either fresh or plastic-embedded tissues from the leaves and roots using the proper reagents. The aim was to trace secondary metabolites of special interest, mostly those crucial for the survival of the environmentally stressed plants.

The histochemical reagents employed for the semithin sections of plastic-embedded tissue (pet) were as follows:

(a) Saturated Sudan black B solution in 70% ethanol [10] for the detection of lipids;
(b) Saturated alcian blue solution in 3% acetic acid [11] for the detection of any stored polysaccharides;
(c) 1% aniline blue black in 70% acetic acid [12] for the histochemical detection of accumulated proteins.

Free-hand sections of fresh tissues, along with 15 μm sections obtained from a cryotome (Leica CM1850, Germany) at −10 °C, were stained with standard histochemical reagents:

(a) Osmium tetroxide [13] for unsaturated lipids;
(b) Concentrated H_2_SO_4_ [14] for sesquiterpenes;
(c) Vanillin/HCl [15] for flavonoids;
(d) Antimony trichloride (SbCl_3_) [16] for terpene-containing steroids;
(e) Dittmar’s reagent [17] for alkaloids;
(f) Potassium bichromate [18] for phenolics;
(g) Alcoholic vanillin-HCl (vanillin test) [19] for phenolic compounds;
(h) Ferric chloride [20] for polyphenols;
(i) 4-Nitrosophenol in concentrated H_2_SO_4_ [21] for monoterpene phenols;
(j) Wagner and Ellram reagents [17] for alkaloids;
(k) DMB (3,4-dimethoxy-benzaldehyde or veratraldehyde) for phenolic tannin precursors [22].

All stains were matched to controls. All glass mounts were observed with an OLYMPUS CX41 optical microscope.

## 3. Results and Discussion

### 3.1. Seed Morphology and Anatomy—Germination

**Figure 2:**
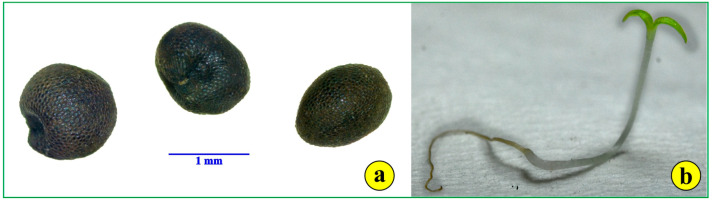
The seeds of *S. jagelii*: (**a**) intact seeds before imbibition; (**b**) a sprout of *S. jagelii*.

**Figure 3:**
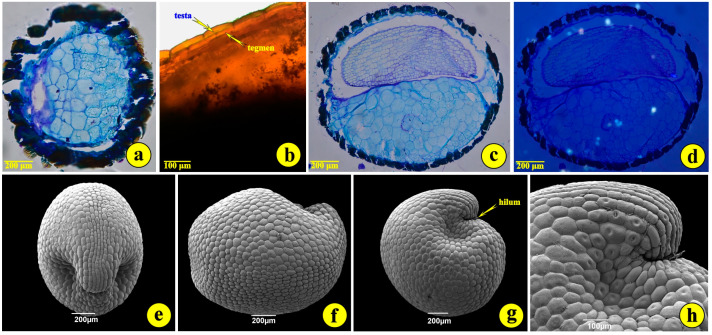
The seed of *S. jagelii*. (**a**–**d**), light micrographs; (**e**–**h**), scanning electron micrographs. (**a**) A tangential section through the seed coat; (**b**) a cross-section of fresh material to demonstrate the seed coat layers; (**c**) a medial section through the endosperm and the embryo; (**d**) the same as (**c**) observed with polarized light to trace any crystalline structures within the embryo. (**e**) The anterior view of the seed; (**f**) the side view of the seed; (**g**) the rear view of the seed; (**h**) details of the hilum.

The number of seeds per capsule, counted from capsules ruptured in the lab, ranged from 7 to 14 seeds. The mean number was 9.4 seeds per capsule. The seeds of *S. jagelii* are more or less globular, with a diameter of 1.0 to 1.5 mm (Figure 2a and Figure 3g). They are bitegmic, with the testa derived from the outer integument, while the tegmen originates from the inner integument (Figure 3b). They present a peculiar deformation on the side of the hilum (Figure 3e,g,h). Their developed testa looks like a surface covered with rather elliptical “tiles” (Figure 3e–h). These are the outer (epidermal) cells of the seed coat. They possess intensely stained, thick, cutinized external cell walls (Figure 3a–c). The embryo undergoes a globular stage in immature seeds, develops into the torpedo stage and bends to display the full cotyledon curvature. The mature embryo is curved at the apex. The suspensor is hardly detectable. The endosperm is of the “cellular type” and consists of large cells occupying most of the seed volume (Figure 3b,c). Observations with polarized light did not reveal any inorganic crystalline structures, as are common in the seeds but not in the leaves and roots of most halophytes (Figure 3d) [23].

The development of the germinated seeds was studied in detail (Figure 2b). The roots grow rapidly to a considerable length, while cotyledons quickly become photosynthetically active (Figure 2b). The highest germination rate was observed at 10 °C, and all seeds germinated in the dark, an indication of total photoinhibition, a “habit” common for plants growing on coastal sand dunes [24,25].

The promotion of seed germination at low temperatures (at 10–15 °C) seems to agree with the environmental conditions, particularly at ambient temperatures during the rainy season [25,26].

### 3.2. Leaf and Root Anatomy

**Figure 4:**
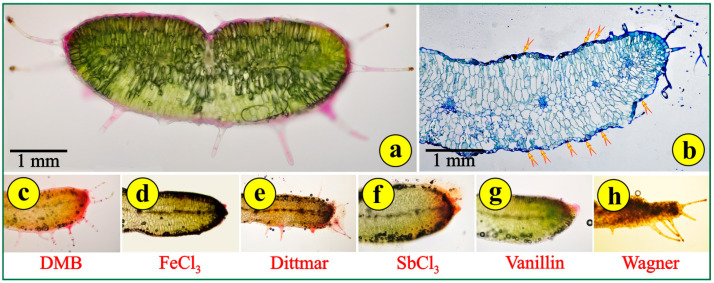
Leaf cross-sections: (**a**) free-hand section; (**b**) section of epoxy-embedded tissue stained with toluidine; arrows point to stomata; (**c**–**h**) application of histochemical reagents to fresh leaf sections; colored areas indicate positive reactions.

Free-hand cross-sections of the fleshy, reddish-green leaves reveal a compact structure with delicate trichomes (Figure 4a). The leaves are dorsiventral but without the typical palisade and spongy parenchyma. The mesophyll cells on the adaxial side are somewhat elongated, while those on the abaxial side are more or less globular (Figure 4a,b). This arrangement simulates the leaf structure of succulents rather than the leaf structure of xerophytes. The cells of the epidermal tissue throughout the leaf surface are rather thin with intensely stained cell walls (Figure 4b). The conductive tissue is far from being developed. There is no mechanical tissue for the support of the conductive bundles, the dispersion of which is rather sparse. Stomata are observed on both sides of the leaf (Figure 4b). Stomata on both sides is a typical feature of some xerophytes, but most Mediterranean plants, especially the very-well-adapted evergreen sclerophylls (maquis) and those that are seasonally dimorphic (phrygana), are hypostomatic. Young leaves have long, pilate (uniseriate multicellular) secretory trichomes (Figure 4a). Their secretory heads are located on the top of multicellular stalks. These trichomes can be observed mostly on the margins of the leaves (Figure 4a,b). Aged leaves are largely free of trichomes. The density of the trichomes on the leaf and the concentrations of alkaloids rapidly decrease with the leaf age. This suggests that the functional role of trichomes is likely to be most important in the early stages of *Saponaria*’s leaf development, when the epidermal tissue has not been completely differentiated [27]. The application of histochemical reagents indicated mild reactions, mostly in the epidermal tissue and the glandular heads of the trichomes, for phenolic tannin precursors (DMB) (Figure 4c); polyphenols (FeCl_3_) (Figure 4d); alkaloids (Dittmar) (Figure 4e); terpene-containing steroids (SbCl_3_) (Figure 4f); flavonoids (vanillin) (Figure 4g); and alkaloids (Wagner) (Figure 4h). These metabolites are common in stressed plants, mainly in their leaves. All other histochemical reagents employed did not cause a reaction.

**Figure 5:**
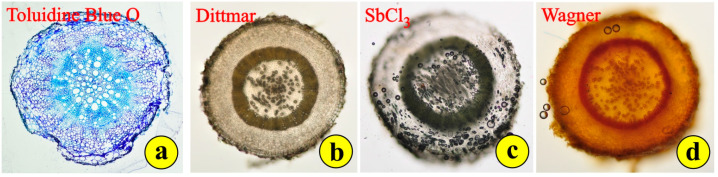
Root cross-sections and histochemistry: (**a**) section of epoxy-embedded tissue stained with toluidine blue; (**b**) fresh tissue stained with Dittmar stain for alkaloids; (**c**) fresh tissue, no reaction with SbCl_3_; (**d**) fresh tissue stained with Wagner, positive reaction for alkaloids.

The roots of *S. jagelii* appear to quickly prepare for the stressing conditions of the arid, salty environment. *In-situ*, the roots are very long and probably grow rapidly downwards to explore deep soil horizons for any signs of water. In cultured plants, after seed germination, it seems amazing how rapidly the root grows and how quickly it passes from the primary to the secondary structure. Very young roots, close to the root tip, demonstrate an elaborate woody structure (Figure 5a). Wide tracheary elements appear in the middle, while more thick-walled sclerenchyma cells appear to clasp the narrow vascular elements. The rays are few and uniseriate. The cells in the cortex are densely accommodated (Figure 5a). The whole structure of the root seems to help the plant penetrate the soil and overcome the difficulties in finding and transporting water. After a series of histochemical investigations, only alkaloids were detected in the root (Figure 5b–d).

### 3.3. Flower and Fruit Anatomy

**Figure 6:**
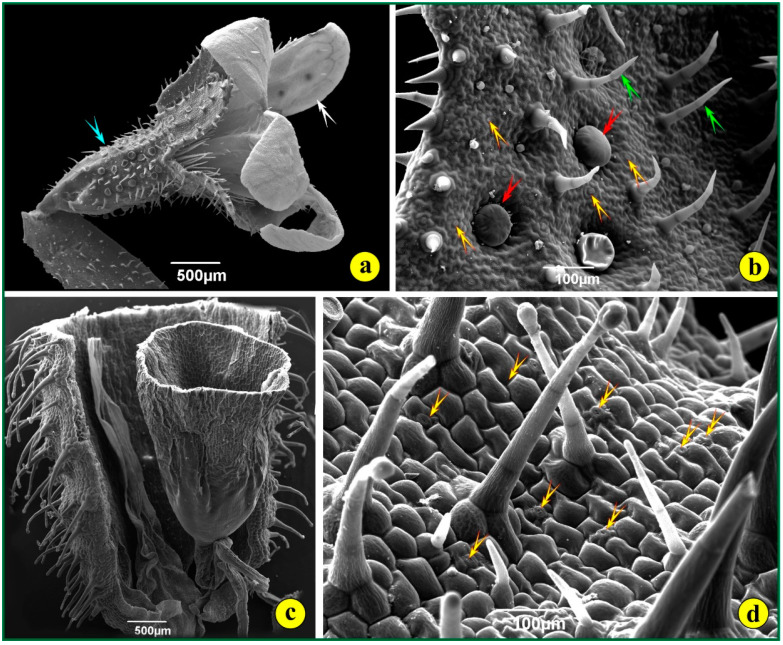
The flower and the fruit of *S. jagelii*. (**a**) A whole mount of the flower. The cyan arrow points to the calyx, while the white arrow points to the petals; the capitate secretory trichomes can be observed on the surfaces of the sepals. (**b**) A part of the abaxial side of a sepal. The red arrows point to the peltate hair, the green ones to the uniseriate, cuspidate, defensive trichomes, and the yellow ones to stomata. (**c**) A scanning electron micrograph of a dissected fruit. The inner, funnel-shaped part is the hard, rather woody endocarp. (**d**) The adaxial side of the exocarp demonstrating glandular pilate trichomes with multicellular stalks. The arrows point to stomata.

The light-red to purple petals of the *S. jagelii* flowers (white arrow in Figure 6a) are surrounded by a hairy calyx (cyan arrow in Figure 6a). The sepals are lined with trichomes. Capitate trichomes are frequent on the abaxial side of the petals. They develop a large head, full of excreted material, which is probably a “call” to pollinators (red arrows in Figure 6b). Uniseriate, (multicellular) tapering, cuspidate defensive trichomes are abundant all over the abaxial side of the sepals as well as on their upper margins (Figure 6a, green arrows in Figure 6b). Numerous stomata of the anomocytic type can also be observed. Among the stomata complexes, a few appear to be diacytic (yellow arrows in Figure 6b,d).

**Figure 7:**
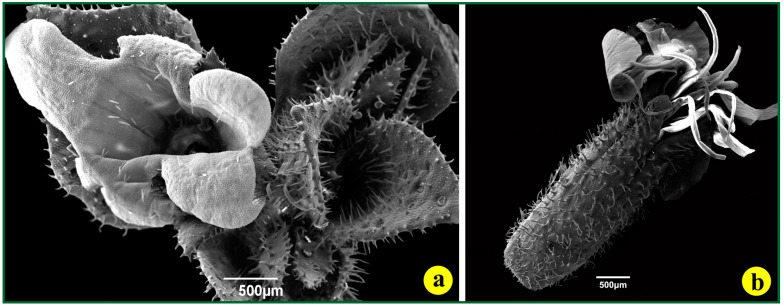
The flower, the leaf primordia and the immature fruit of *S. jagelii*. (**a**) A whole mount of the mature flower and the apical meristem. The slightly hairy petals and the short stamens, deep in the corolla, can be observed on the left side; the leaf primordia, covered with pointed defensive hairs and peltate glandular trichomes, are demonstrated on the right side of the figure. (**b**) A whole young fruit is observed with the stamens and the petals disorganized; the sepals, fully covered with pointed defensive hairs, are still tightly joined.

**Figure 8:**
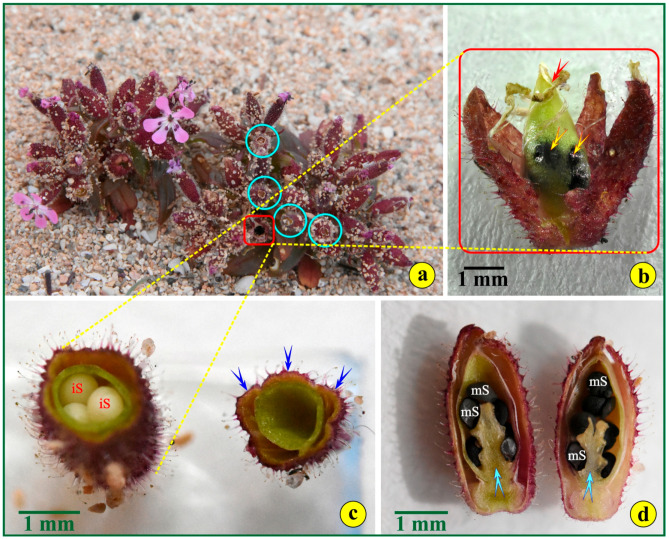
(**a**) An *S. jagelii* cluster of 3–4 individuals with naturally opened capsules (cyan circles): a capsule with seeds in it is indicated by the red square. (**b**) An open capsule: yellow arrows point to the seeds within the endocarp; the red arrow points to the remnants of the pistil. (**c**) Cross-sectioned capsules: immature seeds (iS) are indicated (**left**), while the carpels are marked with blue arrows on the empty capsule (**right**). (**d**) An axial, longitudinal section of the fruit. The cyan arrows point to the placenta; “mS” means “mature seeds”.

The fruit is a capsule (Figure 6c), 15–20 mm long, opening by four ascending, recurved teeth (carpels) (Figure 7a and Figure 8a,c). It has a fleshy, hairy exocarp, a thin mesocarp and a thin-walled, hard endocarp (Figure 6c). Glandular pilate trichomes with multicellular stalks, accommodated on an elevated, rosette-like base, cover the whole surface of the exocarp (Figure 6b,d). Between these trichomes, numerous diacytic stomata can be observed (Figure 6d). The immature fruits are fully covered with pointed defensive hairs (Figure 7b).

Within the fruit (red rectangle in Figure 8a), the mature ovules ripen to kidney-shaped seeds (Figure 8b). Their dark-brown seed coats appear pebbled, lacking any appendages (Figure 8d). In both cross- and longitudinal sections, the seeds, immature (Figure 8c) or fully mature, are attached to an axile, free, central placenta (Figure 8d).

### 3.4. Habitat, Threats and Protection

The annual species of *S. jagelii* has a peculiar ecology. It is characterized, according to the “*Vascular plants of Greece*” [28], as a Therophyte, [“Annuals, completing their life cycle (sometimes several times) within one growing period, surviving the unfavorable period as seeds or seedlings (spring-green, summer-green or overwintering-green ephemerals”)], while the habitat is described as coastal (“Marine waters and mudflats, salt marshes, sand dunes, littoral rocks, halo-nitrophilous scrub”). It thrives on the coastal sand dunes of the small island of Elafonisos, located close to the southern coast of the Peloponnese mainland. The species was reported initially as “Endangered” [3] and then “Critically Endangered” (1998 and 2006) according to IUCN [4]. It is not included in any international convention or national legislation. The plant is indirectly protected, as it falls within the Natura 2000 site GR2540002 “Periochi Neapolis kai Nisos Elafonisos”.

**Figure 9:**
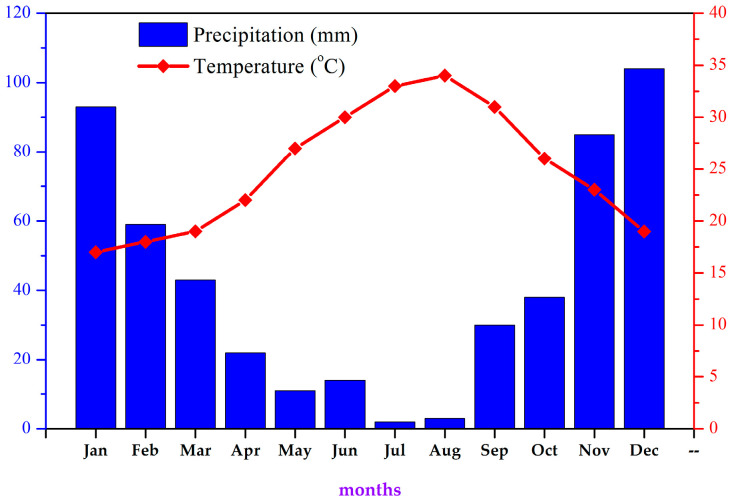
A temperature/precipitation chart for the island of Elafonisos (mean values for 2017–2022), where the natural environment of *S. jagelii* is located.

Under the Köppen–Geiger climate classification, Elafonisos (longitude 22.9594983; latitude 36.4875509) features a hot-summer Mediterranean climate (Csa). Temperatures typically range between 12 °C and 28 °C throughout the year (Figure 9). On rare occasions, they can drop to 3 °C or rise to as high as 36 °C (−1.46% lower than Greece’s averages). The island typically receives an average of 55.88 mm of precipitation and has about 104 rainy days (28.72% of the time) annually (Figure 9). Elafonisos enjoys an average of 4006 h of sunshine throughout the year, and daylight varies from 9 h 41 min to 14 h 35 min per day [29,30]. In this arid, high-salinity environment, a tiny plant species struggles to survive against human disturbances and oncoming climate change.

Specifically, on the island of Elafonisos, *S. jagelii* was found only in two distinct regions. The distance between these two regions is about 2 km. The first region is easily accessed, since it is next to an often-visited beach, where tourists or locals can come across, “face to face”, this critically endangered species. This means that, starting in May, when the number of tourists on the sandy beaches of Elafonisos is increasing rapidly, many of the individuals are probably trampled by cars, motorcycles, and off-road vehicles, subjected to littering by human tourists/visitors, etc. Indeed, during two consecutive years of our investigation and the visits *in-situ* (2022, 2023) [25,26,31], no individuals were found, in contrast to 2019 data [32]. In this region, the population of the species is probably lost, which is the most pessimistic scenario, unless the seed bank remaining buried within the sand is mobilized on time, i.e., within a few years. Bearing in mind that the germination of the seeds occurs under dark conditions, these buried seeds may germinate at some time. During our last visit on 1 April, *S. jagelli* was found to be in mid-flowering season, while the full process of seed production and thus dispersion seems to be completed by the middle to the end of May, when the touristic activities on the island are starting to increase.

The second region, where the main population of fewer than 2000 individuals was found, is more isolated and not easily accessed. Unfortunately, on the sand, we can easily trace the output of touristic motorcycles. In this region, whose area in the formed polygon is less than 2000 m^2^, the population of the species still thrives. Interestingly, a small increase of 5% of the total population was recorded (compared to data from 2022 and 2023). This population polygon consists of two different substrates: one is the already-known coastal dunes, while the other substrate is composed of a really limited sandy area and an area mainly consisting of little pebbles. The mean tendency of the species is to remain as individuals rather than forming clusters; individuals are found in close contact (e.g., 2–3 cm). The distribution of individuals during the two visits was about the same; ~75% of the measured plants did not form clusters. The most interesting note is that during the visit in the second year, a major increase in the individual form was detected in the sandy area of the population polygon in areas closer to the sea. This is considered to be of great importance since lots of litter—mostly dead, semi-decomposed aquatic plants and animals, as well as human-generated garbage, washed ashore by south/north–south winds—cover, many times, a part of the shore on which *S. jagelii* is thriving. This minor increase in geographic range in the areas closer to the sea, where a large number of individuals—not clusters—were detected due to the above-referenced litter, could result in the species being buried under it, posing some new questions about the future of this population. We have not provided any coordinates in order to protect the plant.

It is crucial to point out the direction of the wind in the region. The habitat is strongly affected by west and north-west winds. According to data obtained by the National Observatory of Athens (NOA), these directions prevail only in April, while from May to August, winds in the eastern direction are dominant in this area. Recent research [2] reporting the existence of the plant on Lemnos Island fails to give any information about the size of the population.

Finally, what seems to be of higher importance is that the island of Elafonisos has a rapidly developing tourist industry, accompanied by several so-called “recreational activities” on the beaches. The use of noisy off-road vehicles crossing the dunes and the thousands of visitors trampling the plants have proven to be a major threat, especially during the flowering period. This could result in a further rapid decline in the population and, eventually, cause the extinction of the species.

*S. jagelii* is being cultivated at the Seed Bank of the National and Kapodistrian University of Athens (Greece) and the “I. & A. N. Diomidis Botanical Garden” in Athens (Greece).

## 4. Conclusions

According to “The Red Data Book of Rare and Threatened Plants of Greece” [3], the small plant of *S. jagelii*, thriving in a remote corner of our planet, is facing extinction. We traced this plant and carefully recorded its phenology, as well as the attributes and the peculiarities of its micro-environment. Very sparingly, we collected seeds and the aboveground parts of two plants for further anatomical investigation. The seeds germinated, and the seedlings rapidly developed; their roots were also excised for microscopic investigation.

*S. jagelii* has evolved into a “smart” species concerning its current stress-escaping strategies. The leaves are well equipped for a xeromorphic life, yet they do not possess the typical structure that xerophytes have adapted to thrive in the high temperatures of arid areas of Greece. The leaf epidermis is thin, and the mesophyll is moderately compact, approaching the leaf structure of succulents rather than the leaf structure of xerophytes. The plant is not hypostomatic as most well-adapted Mediterranean species are. The roots appear prepared to explore deep soil horizons and transport water under highly unfavorable conditions. The season of flowering and seed dispersal runs ahead of the unfavorable season. This is also a major advantage, favoring the plant’s ability to withstand a moderate tourist invasion during the peak summer season, since the fruit capsules ripen before the end of May.

The anatomical and ecophysiological observations discussed in the current research might also serve as a tool for exploring the potential of the plant to survive the constantly rising temperatures of climatic change. However, the species might not be prepared to face such rapid changes happening simultaneously in its environment. The highest plant germination rate was observed at as low as 10 °C, while the temperatures on the island range higher than that, between 12 °C and 28 °C, throughout the year (Figure 9), with a probability of an increase due to climate change. Currently, seed production and dispersion seem to occur by the middle to the end of May. The high temperatures will affect not only the germination of the species but also the survival of seedlings, as the threat of touristic activities will be higher, with the tourist season starting earlier in May—a tendency that is already noted in all Greek islands. Visitor arrivals on the island, even in the middle of the COVID-19 pandemic (2020 *, 2021 *), are given above in Table 1 [33].

Considering all these pieces of information, and in agreement with the conservation actions suggested in the IUCN Red List of Threatened Species [4], we suggest the following actions be considered for the protection of the species. We strongly recommend that all vehicles be strictly prohibited from accessing the sand dunes, as this is crucial for the survival of the species. In addition, clearly marked obligate paths, with obvious information panels, must be established. Moreover, the sand beaches must be made inaccessible to visitors during the seed dispersal period of *S. jagelii*, i.e., from early May to early June. During the other months of the touristic period, from July to September, all activities in the area must be mild. After all, there are some other visit-worthy, fantastic sandy beaches on the island (e.g., Simos beach). Another idea focused on raising awareness of both local people and visitors is the creation of plant Micro-Reserves, a protected area with less than 20 ha surface [34]. This continuous monitoring system could be a major tool for the conservation of this rare endemic species and a major help for preserving biodiversity in this NATURA2000 site [35,36].

The authors hope that the new eco-anatomical information added to the current knowledge of the species will facilitate, through better-informed decisions, conservation actions to support *Saponaria jagelii* and protect this fantastic “dwarf” plant.

## Figures and Tables

**Table 1:** Visitor arrivals on the island, 2018 to 2022. The island can be accessed only by boat.

2018	2019	2020 *	2021 *	2022	Months
5.135	5.622	5.436	6.332	5.533	J, F, M
28.957	36.232	15.603	32.971	32.983	A, M, J
106.661	122.234	116.625	124.017	124.187	J, A, S
9.072	9.645	9.772	9.147	13.335	O, N, D
120.868	173.733	147.436	172.467	176.038	total

Asterisks (*) indicate the years of pandemic and lock down.

## Data Availability

Any pieces of data supporting the conclusions of this article will be made available by the authors on request.
